# Supplementary figures and images for: Mode of action of the 2-phenylquinoline efflux inhibitor PQQ4R against Escherichia coli
Source: PeerJ. 2017 Apr 26;5:e3168. doi: 10.7717/peerj.3168 (PMC5433425; doi:10.7717/peerj.3168)

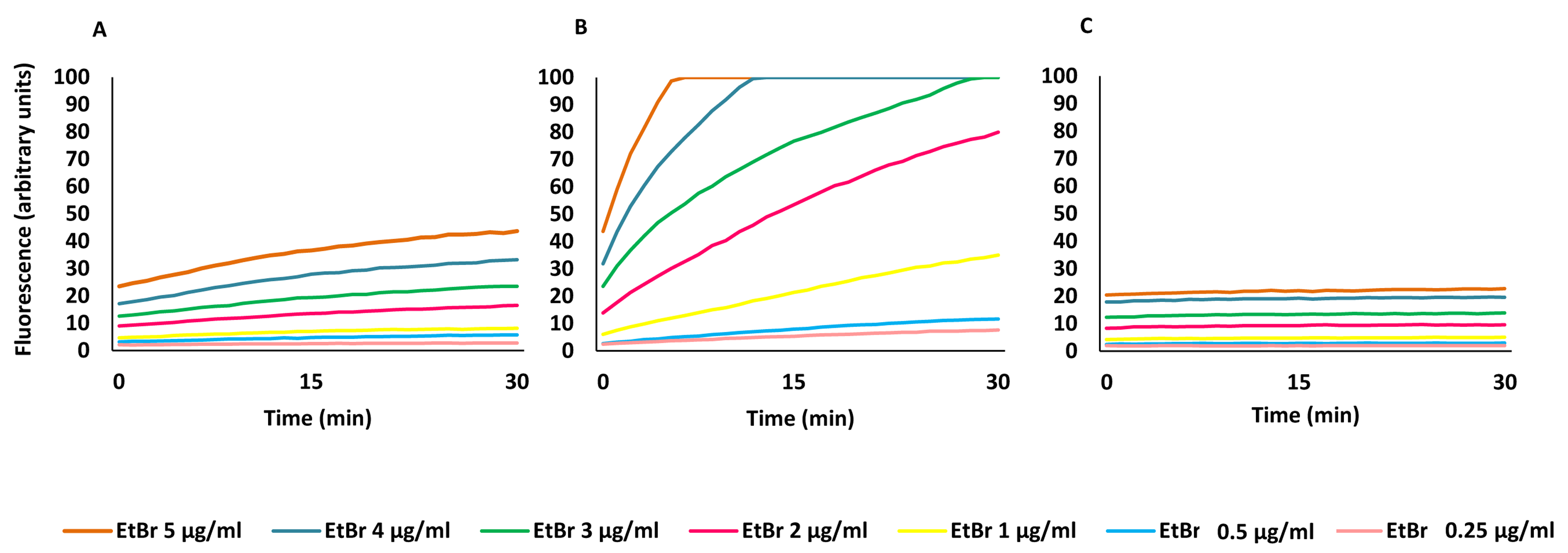

Supplement: Figure S1 [file peerj-05-3168-s001.png]
